# Deletion of the Human Cytomegalovirus US2 to US11 Gene Family Members Impairs the Type-I Interferon Response

**DOI:** 10.3390/v17030426

**Published:** 2025-03-15

**Authors:** Inessa Penner, Nadine Krämer, Julia Hirsch, Nicole Büscher, Hanno Schmidt, Bodo Plachter

**Affiliations:** Institute for Virology and Research Center for Immunotherapy, University Medical Center of the Johannes Gutenberg-University Mainz, 55131 Mainz, Germany; inessa.penner@gmail.com (I.P.); kraemer_nadine@gmx.de (N.K.); julia.hirsch100@gmail.com (J.H.); bueschni@uni-mainz.de (N.B.); hanno.schmidt@uni-mainz.de (H.S.)

**Keywords:** cytomegalovirus, immune evasion, interferon, interferon stimulated genes

## Abstract

Infection of cells with the human cytomegalovirus (HCMV) triggers the expression of interferon-stimulated genes (ISGs). ISGs encode proteins with antiviral functions, such as inhibiting viral replication, promoting cell death of infected cells and enhancing immune responses. HCMV has evolved mechanisms to evade the antiviral effects of ISGs. The viral proteins encoded by the viral genes US7, US8, and US9 have been shown to interfere with interferon induction. US7 to US9 are embedded in a cluster of HCMV genes, termed US2 to US11. The individual members of this gene family interfere on multiple levels with innate and adaptive immune responses to HCMV infection. Using viral mutants with different deletions in US2 to US11, we addressed the question if genes other than US7 to US9 would also influence the IFN responses. Surprisingly, deletion of the complete US2 to US11 gene region led to reduced levels of selected ISGs. Cells infected with viruses in which individual US2 to US11 genes were deleted showed a less pronounced reduction of the selected ISGs. The experiments including RNA-seq analyses indicate that genes of the US2 to US11 gene family have a complex interaction with the IFN-ISG response which is likely regulated on the level of ISG protein stability. As US2–US11 are dispensable for replication in cell culture, the genomic region was frequently used for the insertion of bacterial artificial chromosome vectors in the process of cloning the complete HCMV genome. The results shown here must be considered when viruses derived from BACs with US2–US11 deletions are used and whether appropriate controls must be applied.

## 1. Introduction

The human cytomegalovirus (HCMV) has developed multiple strategies to evade host defence mechanisms [1,2,3,4,5,6,7,8]. Of particular interest in this respect are the proteins encoded by the US2 to US11 gene region. Early work showed that the deletion of these genes abrogated the HCMV-mediated downregulation of MHC-class I expression on the surface of infected cells [9]. Further studies demonstrated that individual US2 to US11 genes interfere with both MHC-class I and MHC-class II antigen presentation [10,11,12,13]. In addition, US7–US9 genes have been reported to affect the innate response to HCMV infection by targeting Toll-like receptors (TLRs), the Mitochondrial Antiviral-Signalling Protein (MAVS) and the Stimulators of Interferon Genes (STING)-mediated signaling pathways [14,15]. Although the genes encoded by US2 to US11 appear to be important for HCMV replication in-vivo, they are non-essential in cell culture [16,17,18]. Thus, this region has been frequently selected for the insertion of bacterial artificial chromosome (BAC) vectors into the genome of HCMV in order to clone the complete genome of HCMV in bacteria and make it accessible for targeted genetic modification [19,20,21].

A viral mutant of HCMV lacking the US7–US16 region has been shown to have an impaired ability to interfere with TLR3 and TLR4 activation [14]. The same mutant is impaired in the antagonism of MAVS/STING-mediated IFN-β expression, controlled by US9 [15]. However, it remained unclear whether the lack of US7 to US9 genes had an impact on the downstream induction of interferon-stimulated genes (ISGs), many of which act as antiviral restriction factors [22]. In addition, the role of other proteins within the US2 to US11 gene region on ISG induction remained unknown. Except for the role of these genes in downregulating MHC-class I and –class II mediated antigen presentation, e.g., US2 has also been reported to selectively target other cell surface proteins for degradation [23].

We thus addressed the roles of US2 to US11 proteins in ISG induction by using a set of viral mutants. Opposed to our expectation, the steady-state levels of selected ISGs were reduced in cells infected with an US2–US11 mutant of HCMV. Similar effects were also seen with mutants in which selected sets of US2–US11 genes were deleted. These results suggest that the effects mediated by US2–US11 genes are interdependent, as the knock-out of single genes or a set of genes may not completely mirror the function of these genes in the context of infection. In addition, the results indicate that the insertion of heterologous genes in the US2–US11 gene region for a phenotypic comparison to wild-type- (wt-) virus may be biased by a different induction of the interferon response.

## 2. Materials and Methods

### 2.1. Cells, BAC-Cloning, Western Blots, and Viruses

Primary human foreskin fibroblasts (HFF) were cultured as described before [24]. All HCMV strains used in this analysis were derived from bacterial artificial chromosome (bacmid) clones. The HCMV strain BADwt was obtained from Thomas Schenk, (Princeton University, Princeton, NJ, USA) [25]. The strains BAD-ΔUS2-11 [26], BAD-ΔUS2,3,6,11 (KB6), BAD-ΔUS2,3,6 (KB9), ΔUS3,6,11 (KB13) [24], and BAD-ΔUS2,6,11 (KB7) [27] have been described before. For the generation of the novel viral strain BAD-ΔUS7-10, a PCR fragment was generated using the plasmid pgalK [28] as a template with flanking sequences to target the US7–US10 gene region of pAD/cre (the bacmid of strain BADwt). This sequence was cloned into a plasmid vector. The galK-encoding DNA fragment was gel purified and subsequently transformed into bacteria, containing pAD/cre. Recombinant bacmids were positively selected, using the GalK selection procedure [28]. The bacmid DNA from bacterial clones was tested by PCR for correct insertion and was subsequently used for transfection and viral reconstitution in HFF. Western blots were performed as described in [29].

### 2.2. Infection of HFF

The copy number of viral genomes detectable in HFF at 6 h after infection was used as a measure for the virus concentration in culture supernatants used for subsequent experiments. For this, viral stocks were first generated by infecting HFF with the desired viral strain. When all cells showed the typical cytopathic effect of HCMV infection, the cell culture supernatants were collected and spilt-delivered into cryotubes. These stocks were stored at −80 °C until further use. For the determination of virus concentration in these stocks, single cryotubes were rapidly thawed and applied to HFF in serial dilutions. For this, HFF were seeded in 10 cm^2^ dishes the day before. The culture supernatants containing the HCMV strains of interest were then applied in serial dilutions to these HFF (10 µL, 50 µL, 100 µL and 500 µL). 6 h post infection (h.p.i.), HFF were washed two times with DPBS, collected, adjusted to 1 × 10^6^/mL in 1 ×DPBS and stored at −20 °C until analyzed by PCR. For this, the viral DNA was isolated out of 1 × 10^5^ thawed cells with the High Pure Viral Nucleic Acid Kit (Roche LifeScience, Munich, Germany) according to the instructions of the manufacturer. 5 µL of isolated DNA was mixed with 45 µL master mix per tube for qPCR. As a non-template control, ddH_2_O was used and serial diluted standard DNA from HCMV cosmid pCM1049 was taken for calibration [30]. Three technical replicates were evaluated, using the 7500 Real-Time PCR system (Thermo Fisher Scientific, Waltham, MA, USA). Genome copies were analyzed by the 7000 System SDS software V.1.2.3. Copy numbers of the viral genome per cell were determined by the mean specific CT value in relation to the standard serial dilutions.

### 2.3. RNA-Seq Analysis

For total RNA isolation 1.5 × 10^6^ HFF were infected with 40 genomes per cell of BADwt, BAD-ΔUS2-11 or BAD-ΔUS7-10. After 24 h, RNA was extracted using the Quick-RNA™ Microprep Kit (Zymo research, Freiburg, Germany). Subsequently, the RNA samples from three independent isolations were assessed for quality utilizing an Agilent 2100 Bioanalyzer Eukaryote total RNA assay (Agilent, Santa Clara, CA, USA). The generated RNA-Seq libraries were subjected to sequencing by a commercial provider (StarSEQ, Mainz, Germany), employing a paired-end strategy to generate 150 bp sequence reads on an Illumina NextSeq 2000 system. RNA-Seq data analysis was accomplished using the DRAGEN pipeline v.4.0.3 [31] including the DRAGEN RNA-Seq spliced aligner, duplicate marking, gene fusion detection, and gene expression quantification modules. The latter runs DESeq2 [32] on Salmon [33] quantification files generated within the pipeline. A gene was considered differentially expressed if the adjusted *p*-value between the groups was <0.05 with no further filtering for log2 fold changes.

## 3. Results

### 3.1. Deletion of Immune Evasion Genes US2–US11 Leads to Impairment in the Induction of ISGs

**Figure 1:**
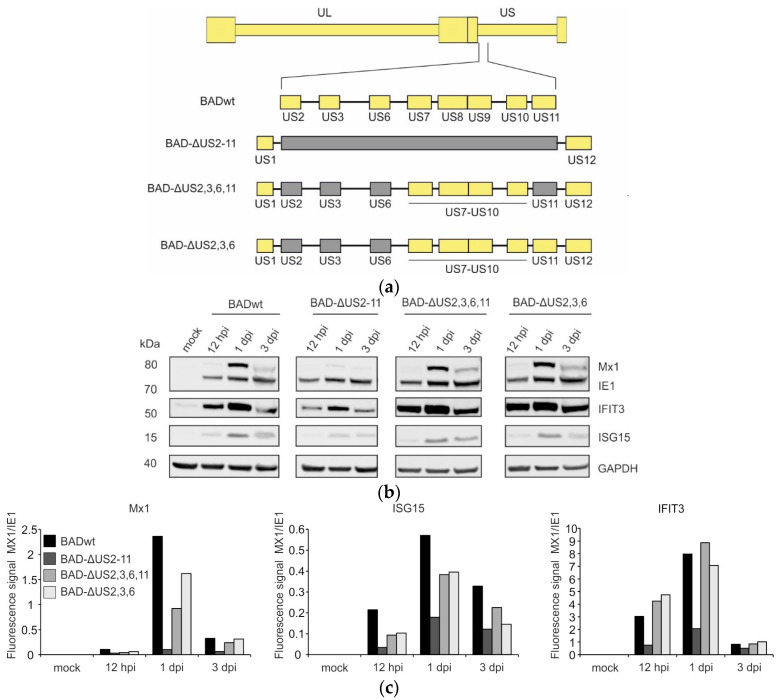
Western blot analysis of the expression of the ISGs Mx1, IFIT3, and ISG15 in HFF, infected with 40 genome copies per cell of the different US2–US11 deletion variants of HCMV. (**a**) Schematic representation of the genomic structure of the different viruses. (**b**) Western blot analysis of lysates from HFF, infected with the indicated viruses for different times after infection. The molecular masses are shown on the left and the proteins targeted by specific antibodies are shown on the right. (**c**) Quantification of IE1, MX1 IFIT3 and ISG15 expression in HFF infected with the mutants compared to HFF infected with wt-virus. The band density of each protein was quantified by the Image Studio™ Lite software (Version 5.0). Staining for GAPDH was used as control. Comparisons between BADwt and BAD-ΔUS2-11 were performed multiple times with comparable results. Analyses using BAD-ΔUS2,3,6,11 and BAD-ΔUS2,3,6 were performed once.

The genomic region of HCMV consisting of the genes US2–US11 is important for the subversion of both innate and adaptive immune responses. Particularly, US7, US8, and US9 have been described to specifically interfere with the induction of interferon responses [14,15]. pUS7 and pUS8 have been identified to target TRL3 and TRL4 for degradation, whereas pUS9 targets MAVS- and STING-mediated signaling pathways. The phenotype of impairment of interferon responses by these genes was verified by using an HCMV US7–US16 deletion mutant [14,15]. We were interested to investigate if there were additional genes in the US2–US11 gene region that would interfere with the interferon response. In an initial experiment, we made use of the viral mutant BAD-ΔUS2-11 which is deficient of all genes in the US2–US11 region (Figure 1a) [26]. The mutants BAD-ΔUS2,3,6,11 and BAD-ΔUS2,3,6 are devoid of genes known to interfere with the MHC-class I antigen presentation pathway (Figure 1a) [24]. Human foreskin fibroblast cells (HFF) were infected with these viruses and the respective parental strain BADwt [25]. Infected-cell lysates were obtained at different times after infection and were subjected to Western blot analysis (Figure 1b). All three selected ISGs namely Mx1, IFIT3 and ISG15 were induced after infection with BADwt. Surprisingly, the level of ISG induction was markedly reduced in BAD-ΔUS2-11 infected cells, compared to BADwt-infected cells. This result was reproduced in several independent experiments. An identical result was obtained by using the virus JH10 [34], an US2–US11 deletion mutant of BADwt, generated independently. A subtly reduced Mx1 and ISG15 induction was seen after infection with BAD-ΔUS2,3,6,11 and BAD-ΔUS2,3,6. Opposed to this, IFIT3 levels were slightly increased compared to the levels seen after BADwt infection, using the latter viruses for infection. Taken together, these results show that the deletion of the entire US2–US11 gene region of HCMV leads to the downregulation of the steady-state levels of the ISGs Mx1, IFIT3 and ISG15. Interestingly, IFIT3 levels were elevated in cells that were infected with viruses that were deficient only in the MHC-class I evasion genes. This showed that the lack of these genes had different effects on the levels of different ISGs.

### 3.2. Deletion of Immune Evasion Genes US7–US10 Impairs the Induction of ISGs

**Figure 2:**
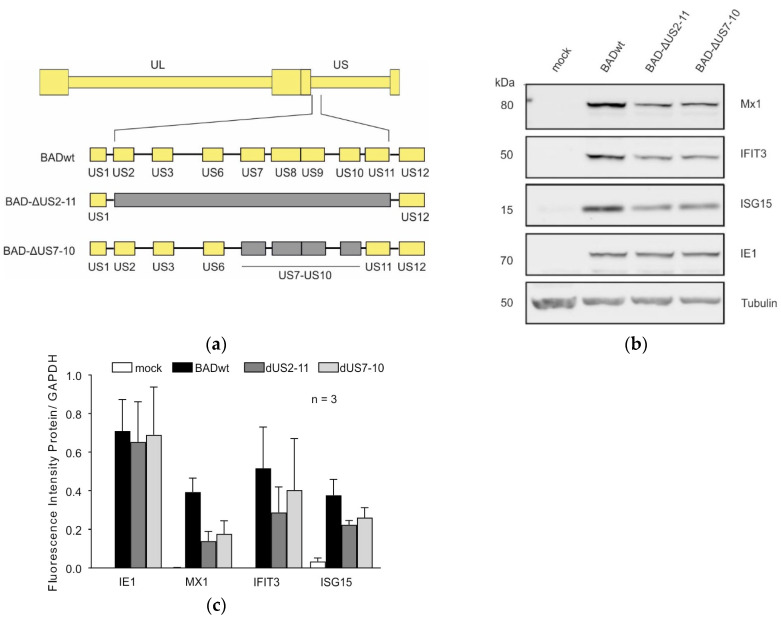
Induction of ISG-expression in dependence on the US2–US11 gene region. (**a**) Schematic representation of the genomes of the viruses used. Yellow bars represent unaltered HCMV genes, grey bars represent deleted genes. (**b**) HFF were infected with 40 genomes per cell of wild-type (wt, strain AD169) HCMV, a mutant lacking the US2–US11genes (ΔUS2-11), a mutant lacking the US7–US10 genes (Δ US7-10), or left uninfected (mock). After 24 h of infection, cells were harvested and subjected to Western blot analysis of endogenous Mx1, IFIT3 and ISG15 protein levels. Expression of the viral IE1 protein was used to monitor equal infection levels. The molecular masses are shown on the left and the proteins targeted by the antibodies used are shown on the right. (**c**) Quantification of IE1, MX1, IFIT3, and ISG15 expression in HFF infected with the mutants compared to HFF infected with wt virus. The band density of each protein was quantified by the Image studio Lite software (Version 5.0). Student’s *t*-test was performed for statistical analysis. Error bars represented mean  ±  SEM of three independent experiments.

The reported influence of the genes encoded by US7, US8, and US9 prompted us to investigate the impact of the deletion of US7–US11 on ISG induction. A virus lacking US7–US11 was generated in the background of BADwt by the BAC technology (BAD-ΔUS7-10; Figure 2a). HFF were infected with this virus and with BADwt and BAD-ΔUS2-11 for control. A Western blot analysis was performed on lysates of infected cells from 1 day after infection (Figure 2b). A reduction of the steady-state levels of Mx1, IFIT3 and ISG15 was seen in both BAD-ΔUS7-10- and BAD-ΔUS2-11-infected cells, compared to BADwt-infected cells (Figure 2c).

### 3.3. Induction of IFIT3 and ISG15 Does Not Require Interferon Signaling

**Figure 3:**
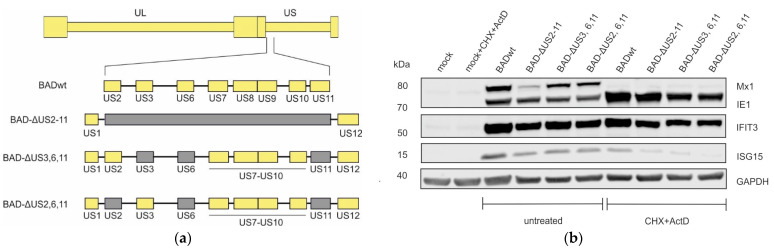
Induction of ISG-expression in dependence on interferon signaling. (**a**) Schematic representation of the genomes of the viruses used. Yellow bars represent unaltered HCMV genes, grey bars represent deleted genes. (**b**) HFF were infected with 40 genomes per cell of the indicated viruses or left uninfected (mock). Cells were either treated by adding cycloheximide (CHX) to the medium for 9 h to block translation or were left untreated. After 9 h, the medium was replaced by a medium, containing actinomycin D (ActD) to block transcription. Cells were collected after 13.5 h of ActD treatment and subjected to Western blot analysis of the endogenous MX1, IFIT3 and ISG15 protein levels. Expression of the viral IE1 protein was used to monitor equal infection levels. An antibody against GAPDH was used to control for equal amounts of loaded cells. The molecular masses are shown on the left and the proteins targeted by the antibodies used are shown on the right. The experiment was performed once.

The proteins encoded by US7, US8, and US9 were shown to interfere with the induction of interferons [14,15]. We wanted to investigate whether the effects afforded by the mutants used in this study on ISG expression were interferon dependent. Thus, cells were infected with the indicated viruses (Figure 3a) in the presence of cycloheximide (CHX) to reversibly block viral and cellular protein translation. After 9 h, the culture medium was replaced by medium, containing actinomycin D (ActD) to block transcription. Under these conditions, infected cells were permissive for the expression of interferons but were blocked for the expression of the downstream ISGs. Cell lysates were probed with antibodies against Mx1, IFIT3, and ISG15. The induction of Mx1 was abrogated in CHX-ActD treated cells, showing that the induction of this protein following HCMV infection depended on the expression of interferons and on interferon signaling (Figure 3b). Surprisingly, both IFIT3 and ISG15 induction appeared to be induced by HCMV independent of interferon signaling. Further to that, the slight reduction in the steady-state levels of these proteins, as seen before, was retained. This suggests that the reduction of the ISG-levels caused by US2–US11 genes was mediated at a level downstream of the interferon-receptor mediated induction of ISGs.

### 3.4. Modulation of ISG-Levels by US2–US11 Requires Viral Gene Expression

**Figure 4:**
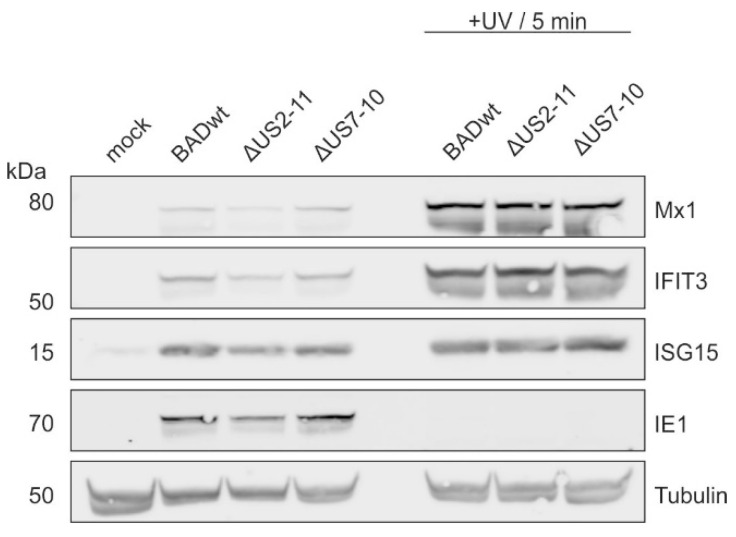
Western blot analysis of the induction of ISG-expression by HCMV infection in the absence of viral gene expression. HFFs were infected with 40 genomes per cell of the indicated viruses. A fraction of each virus was irradiated by ultraviolet (+UV) light to block viral gene expression. The molecular masses are shown on the left and the proteins targeted by the antibodies used are shown on the right. The experiment was performed once.

Infection of fibroblast cells with UV-inactivated HCMV leads to the induction of an ISG response which may be mediated by either the interaction of the virus with its receptor on the cell surface or via regulatory structural proteins delivered by virus particles [35,36]. To address if the differences seen between wt-HCMV and US2–US11 mutants depended on different interaction of the viral particles with target cells, HFF were infected with viruses exposed to ultraviolet light (UV) to prevent viral gene expression. Cell lysates collected at 1 day of infection were analyzed by Western blot (Figure 4). No differences were found in the induction of Mx1, IFIT3 or ISG15 as opposed to what was seen before following infection with replication competent viruses. This indicated that viral gene expression was required for the development of the different phenotypes with respect to ISG levels. The induction of ISGs following infection with UV-irradiated viruses was stronger, compared to the infection with untreated viruses. This can be explained by the effects of UV radiation on virus gene expression. Damaging the viral genome prevents the virus from replication and abrogates its interference with the host interferon response (reviewed in [5,37,38]), leading to higher levels of ISG expression. The lack of IE1-expression showed that viral genes, including those known to interfere with the interferon response were not expressed. Taken together, these results demonstrated that the modulation of the ISG-levels by the US2–US11 proteins requires viral protein expression.

### 3.5. Modulation of ISG Induction by US2–US11 Is Not Caused by Differential Gene Expression

**Figure 5:**
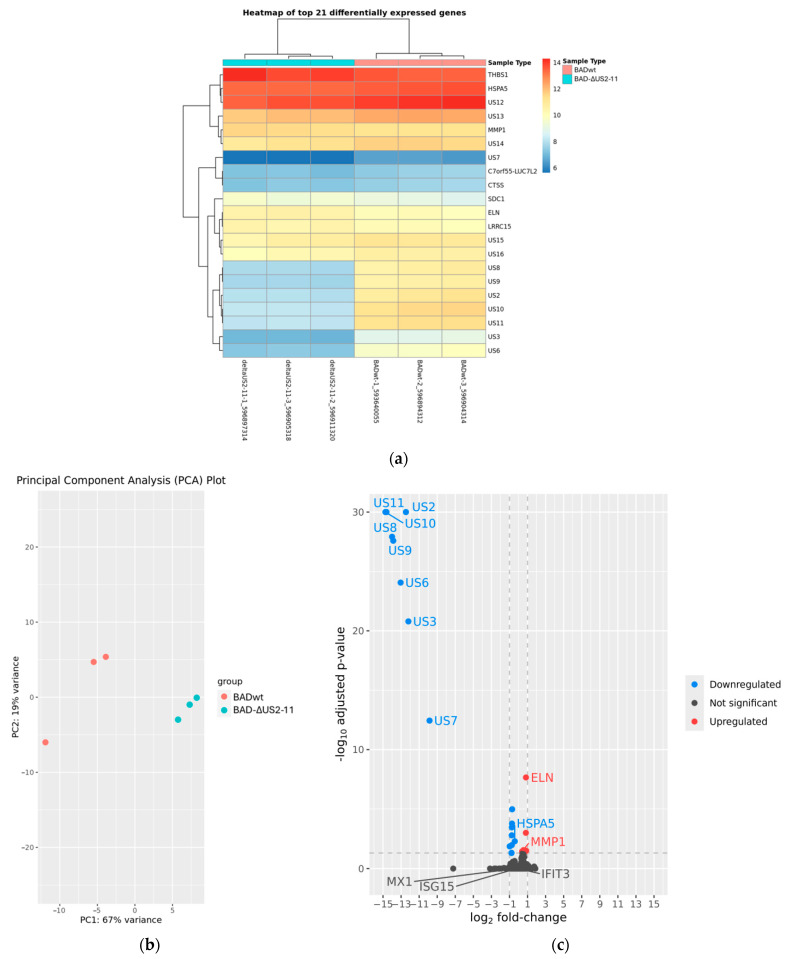
RNA-Seq analysis of BAD-ΔUS2-11 in comparison to BADwt. (**a**) Heatmap with expression values for the six samples for all genes with significant signs of differential expression. (**b**) Principal component analysis for the six samples. (**c**) Volcano plot of differential gene expression analysis. Each point represents one gene with the x-axis showing the log_2_ fold change (log2FC) and the y-axis indicating the –log_10_ adjusted *p*-value (padj). Genes significantly up- and downregulated in the modified virus are highlighted in distinct colors, while non-significant genes are shown in dark grey. Extremely small, adjusted *p*-values (i.e., those below 1E-30) were capped at 1E-30 to avoid distortion of the plot. The dashed line at 0.5 padj denotes the threshold used to determine significance, the dashed line at 1/−1 log2FC is for orientation only. Genes labeled in the plot include the deleted viral genes, the three interferon-stimulated genes discussed in the text (MX1, IFIT3, ISG15), and the three genes identified as differentially expressed in both comparisons (ELN, HSPA5, MMP1).

**Figure 6:**
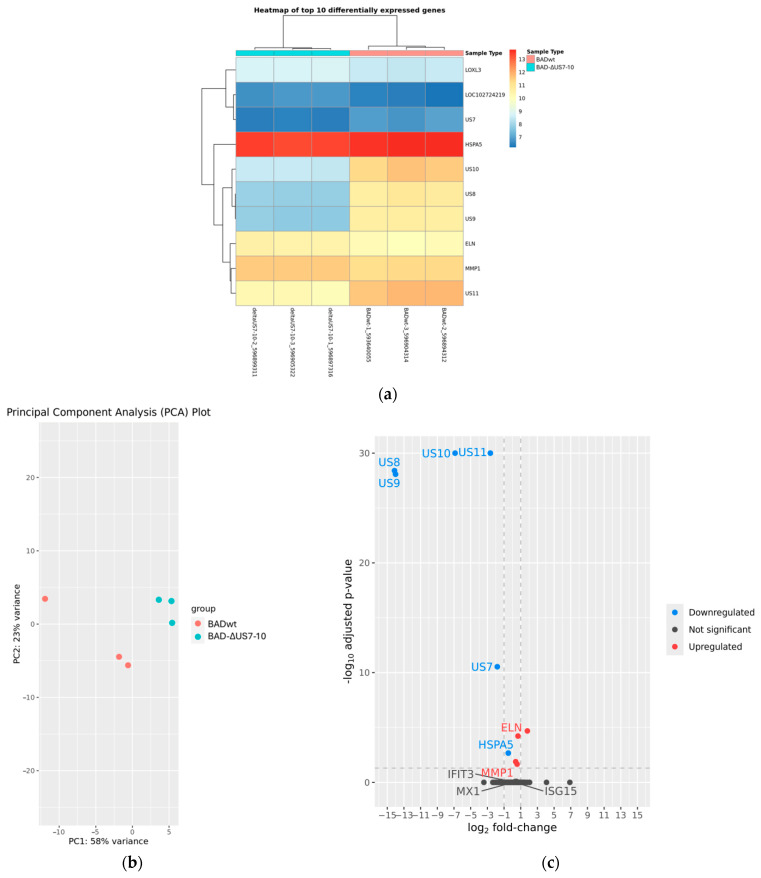
RNA-Seq analysis of BAD-ΔUS7-10 in comparison to BADwt. (**a**) Heatmap with expression values for the six samples for all genes with significant signs of differential expression. (**b**) Principal component analysis for the six samples. (**c**) Volcano plot of differential gene expression analysis. Each point represents one gene with the x-axis showing the log_2_ fold change (log2FC) and the y-axis indicating the –log_10_ adjusted *p*-value (padj). Genes significantly up- and downregulated in the modified virus are highlighted in distinct colors, while non-significant genes are shown in dark grey. Extremely small, adjusted *p*-values (i.e., those below 1E-30) were capped at 1E-30 to avoid distortion of the plot. The dashed line at 0.5 padj denotes the threshold used to determine significance, the dashed line at 1/−1 log2FC is for orientation only. Genes labeled in the plot include the deleted viral genes, the three interferon-stimulated genes discussed in the text (MX1, IFIT3, ISG15), and the three genes identified as differentially expressed in both comparisons (ELN, HSPA5, MMP1).

Several viral proteins are known to interfere with the interferon response and the induction of ISGs. To test whether different patterns of viral RNA synthesis correlated with the differences seen in ISG induction, RNA-Seq analyses were performed. HFF were infected with either BADwt, BAD-ΔUS2-11, or BAD-ΔUS7-10 for 24 h. RNA from three independent biological replicates per treatment was sequenced on an Illumina system with 28 million reads per sample. Differential expression analyses using the DRAGEN pipeline were conducted for the comparisons BADwt against BADΔUS2-11, BADwt against BAD-ΔUS7-10, and BADΔUS2-11 against BAD-ΔUS7-10. These analyses confirmed the successful deletion of the respective viral genes (Figure 5a and Figure 6a). Especially the knockout of US7, US8, and US9 genes, known to interfere with interferon induction was verified in both BAD-ΔUS2-11 and BAD-ΔUS7-10. Also, a clear separation of samples based on their affiliation with the treatment groups was evident (Figure 5b and Figure 6b). However, the expression levels of only very few host cell genes were affected by the aberrant configuration of the respective viruses (Figure 5a,c and Figure 6a,c).

In cells infected with BAD-ΔUS2-11, eight host genes (THBS1, HSPA5, MMP1, C7orf55-LUC7L2, CTSS, SDC1, ELN, LRRC15) were differentially expressed compared to cells infected with wild-type virus (Figure 5a). In cells infected with BAD-ΔUS7-10, five host genes (LOXL3, LOC102724219, HSPA5, ELN, MMP1) were differentially expressed compared to cells infected with wild-type virus (Figure 6a). This means that three genes showed significantly different transcript levels in both comparisons, and with the same direction for all three cases (HSPA5 lower in host cells infected with modified virus, ELN and MMP1 higher in host cells infected with modified virus). The transcript levels of the ISGs, whose protein levels were shown to be altered in the Western blots, were not significantly different in the host cells that were infected with the modified virus strains compared to the host cells that were infected with the wild-type virus. In fact, the adjusted *p*-values for the differential regulation of MX1, IFIT3, and ISG15 are 0.9999 for BAD-ΔUS7-10 and BAD-ΔUS2-11, respectively.

## 4. Discussion

The proteins encoded by US2 to US11 of HCMV have been extensively studied to understand the molecular mechanisms of their attenuation of antiviral immune responses and their interference with antiviral restriction factors [9,10,11,12,13,14,15,22,39]. Many of these proteins act by promoting the degradation of their targets. US7 and US8 bind to TLR3 and TLR4 and destabilize these proteins, thereby interfering with interferon signaling [14]. US2 degrades MHC-class I molecules but also downregulates additional cellular targets by recruiting the cellular E3 ligase TRC8 to direct the proteasomal degradation [23,40,41]. We addressed the question, if US2 to US11 genes had an influence on the expression of restriction factors like ISGs that are expressed via engagement of the interferon-ß receptor with its cognate ligand in HFF. The infection with a US2 to US11 HCMV deletion mutant surprisingly led to a reduction of ISG levels, compared to the parental strain. The infection with viral strains that were only defective in the expression of MHC immune evasion genes US2, US3, US6, and US11 still showed some reduction in the steady-state levels of ISGs Mx1 and ISG15, but also showed slightly elevated levels of IFIT3, compared to the wt-control virus (Figure 1). These former findings can be explained by the fact that these viral strains expressed US7 to US9 which had been shown to interfere with the induction of the interferon pathway [14,15]. However, the infection with a US7 to US10 deleted virus led to the downregulation of ISGs, comparable to a US2 to US11 deleted virus. This shows that the deletion of the genes known to interfere with interferon signalling could not rescue ISG expression to wt levels. Taken together these results indicate that genetic modification within the US2 to US11 gene region interferes in some way with the ability of the cell to respond to HCMV infection. The exact mechanism for this is unclear at this point and requires further analysis. However, this should be considered when using BAC-derived HCMV strains that carry US2 to US11 deletions. Early studies had shown that the US2 to US11 genes were dispensable for viral replication in cell culture [16,17,18]. This rendered the region a favourite location for the insertion of bacterial artificial chromosome vectors in the process of cloning the complete HCMV genome [19,20,21]. Multiple studies have been performed with such viruses, addressing many different issues of HCMV biology, unrelated to the function of US2 to US11. The deletion of US2 to US11 may bias studies in which US2 to US11 competent strains are compared to strains that are genetically altered in this region. The results shown here indicate that appropriate controls are required when using BAC-derived viruses that contain the vector with the US2 to US11 region.

Interferon induction by HCMV infection may be influenced by the interaction of the virus with its cognate receptor or by the introduction of viral proteins into cells [35,36]. Thus, differences between viral strains related to the protein composition of viral particles or particle to infectivity ratios may account for differences in ISG induction. However, the differences between strains were abrogated by exposing cells to UV-inactivated virus. This showed that neither differences in the interaction of the viral particles with the cell nor differences in the amount of particle-associated proteins delivered into cells were responsible for the phenotypic differences. Consequently, viral gene expression was required for the differences seen in the steady-state levels of ISGs.

RNA-Seq analyses were performed to test whether the differences in ISG induction were the consequence of alterations in gene expression. These analyses confirmed the excision of the respective genes from the viral genomes. In BAD-ΔUS2-11 the viral genes US2, US3, US6, US7, US8, US9, US10, and US11 showed no transcription at all. In BAD-ΔUS7-10 the viral genes US8 and US9 showed no transcription at all and the genes US7 and US10 showed strongly and significantly reduced transcript levels. This confirms the findings in the experiments described above and underlines the coherence of the results overall.

In the two experiments, only eight and five host genes, respectively, were found to be significantly differentially expressed between cells infected with wild-type virus and cells infected with modified viruses. Therefore, we can assume that the modification of the virus only marginally altered the response of the host cells to the viral infection on the transcriptome level. Such stable transcriptome patterns increase the confidence in the minimally invasive nature of the virus modifications performed and rule out a broad, unspecific disturbance of the host cell gene expression. When comparing the expression profiles of cells infected with BAD-ΔUS2-11 and cells infected with BAD-ΔUS7-10, only one host gene showed significantly different transcript levels (EPSTI1, Epithelial Stromal Interaction 1 (Supplementary Figure S1). This suggests that the two modified virus strains have virtually no difference in their effect on the host cells at the transcriptome level.

Among the differentially expressed genes, three genes were statistically significantly regulated in both experiments: Heat Shock Protein Family A (Hsp70) Member 5 (HSPA5), Elastin (ELN), and Matrix Metallopeptidase 1 (MMP1). Among these, HSPA5 has a plausible functional link to a response to viral infection, as it is involved in the general cellular stress response and plays a role in cellular apoptosis. HSPA5 showed lower transcript levels in the cells that were infected with the viruses with introduced deletions in both cases. It is therefore quite possible that the coherent downregulation of HSPA5 upon treatment with the modified virus strains is a directed response of the host cells to an altered interaction with the virus. However, at this point it is unclear what the basis of this may be. In addition, the cells infected with BAD-ΔUS2-11 showed significantly reduced levels of transcripts for Cathepsin S (CTSS), a proteinase involved in the degradation of antigenic proteins for presentation on MHC class II molecules. This may be another candidate with potential for a specific response to a modified virus with yet unclear mechanism. No additional genes with plausible links to viral infections were differentially regulated in the BAD-ΔUS7-10 treatment or between the two treatments. These findings include the fact that the interferon-stimulated genes under scrutiny were not differentially expressed in cells infected with any of the modified viruses. This indicates that the differences in steady-state levels of ISGs Mx1, IFIT3, and ISG15 proteins probably were the result of changes in translation efficiency, protein stability or similar processes.

We found no differences in ISG mRNA levels between wildtype and modified viruses but reduced protein levels of ISG products. This may be based on protein stability or translation efficacy. The latter could be tested by analyzing the translatome by ribosome profiling (Ribo-Seq; [42,43,44]). This would reveal whether the ISG mRNAs are underrepresented in the translation process and/or if previously unknown HCMV translation products can be found. This may also include micro RNAs (miRNAs). An immune evasion mechanism has already been described for a miRNA of the unique long (UL) region of the HCMV genome [43], and three miRNAs have been detected in HCMV between US3 and US6 [45]). These miRNAs may well be involved in post-transcriptional gene regulation, most likely through the mechanism of translational repression via the RNA-induced silencing complex RISC [46]. However, translatome and miRNA profiling will be subject to future studies.

One limitation of this study was that only derivatives of a laboratory strain (BADwt, derived from Ad169) were available for analysis. It would be interesting if a similar impact on ISG-levels would be seen when using clinically related strains such as TB40. These latter viruses carry additional genes which are absent from laboratory strains. A TB40 BAC-clone has been generated carrying the vector at a genomic location outside of US2–US11 [47]. It would be instructive to generate a similar set of viral mutants as used here in BADwt in the TB40 background. This is, however, beyond the scope of this study and must be addressed in future work. This will also bear the option to more specifically address the role of US2–US11 genes in the regulation of ISG-levels in infected cells.

Overall, our results suggest that differences in protein stability may play a role in the modulation of ISG levels by the US2–US11 genes. An interesting detail was that deletion of the entire US2–US11 gene region (BAD-ΔUS2-11) leads to a reduction in ISG protein levels, whereas the more confined deletion of the US7–US10 region (BAD-ΔUS7-10) had a smaller effect. This suggests that multiple genes within the US2–US11 region may be cooperatively involved in the regulation of ISG levels. Although this hypothesis was not directly tested experimentally, the Western blot data and RNA-Seq analysis indirectly support this interpretation. The unchanged transcript levels of ISGs in combination with the reduced protein levels suggest post-transcriptional regulation. Previous studies on HCMV have shown that viral proteins can influence the stability of cellular proteins [48]. Our observations extend this understanding and suggest an interplay between the US2–US11 gene products and cellular processes that may influence ISG protein levels via mechanisms such as protein degradation or reduced translation. Further studies could elucidate which specific interactions between the US gene products and cellular signaling pathways cause the observed effects.

## Data Availability

The RNA-Seq datasets are available in the EMBL Nucleotide Sequence Database (ENA) repository under the accession number PRJEB85447.

## Supplementary Materials

The following supporting information can be downloaded at: https://www.mdpi.com/article/10.3390/v17030426/s1, Supplementary Figure S1—BAD-ΔUS2-11_vs_BAD-ΔUS7-10.
