# Supplementary material for: Deletion of the Human Cytomegalovirus US2 to US11 Gene Family Members Impairs the Type-I Interferon Response
Source: Viruses. 2025 Mar 15;17(3):426. doi: 10.3390/v17030426 (PMC11945591; doi:10.3390/v17030426)
Supplement: Supplementary file 1 [file viruses-17-00426-s001.zip › viruses-3484916-supplementary.pdf]

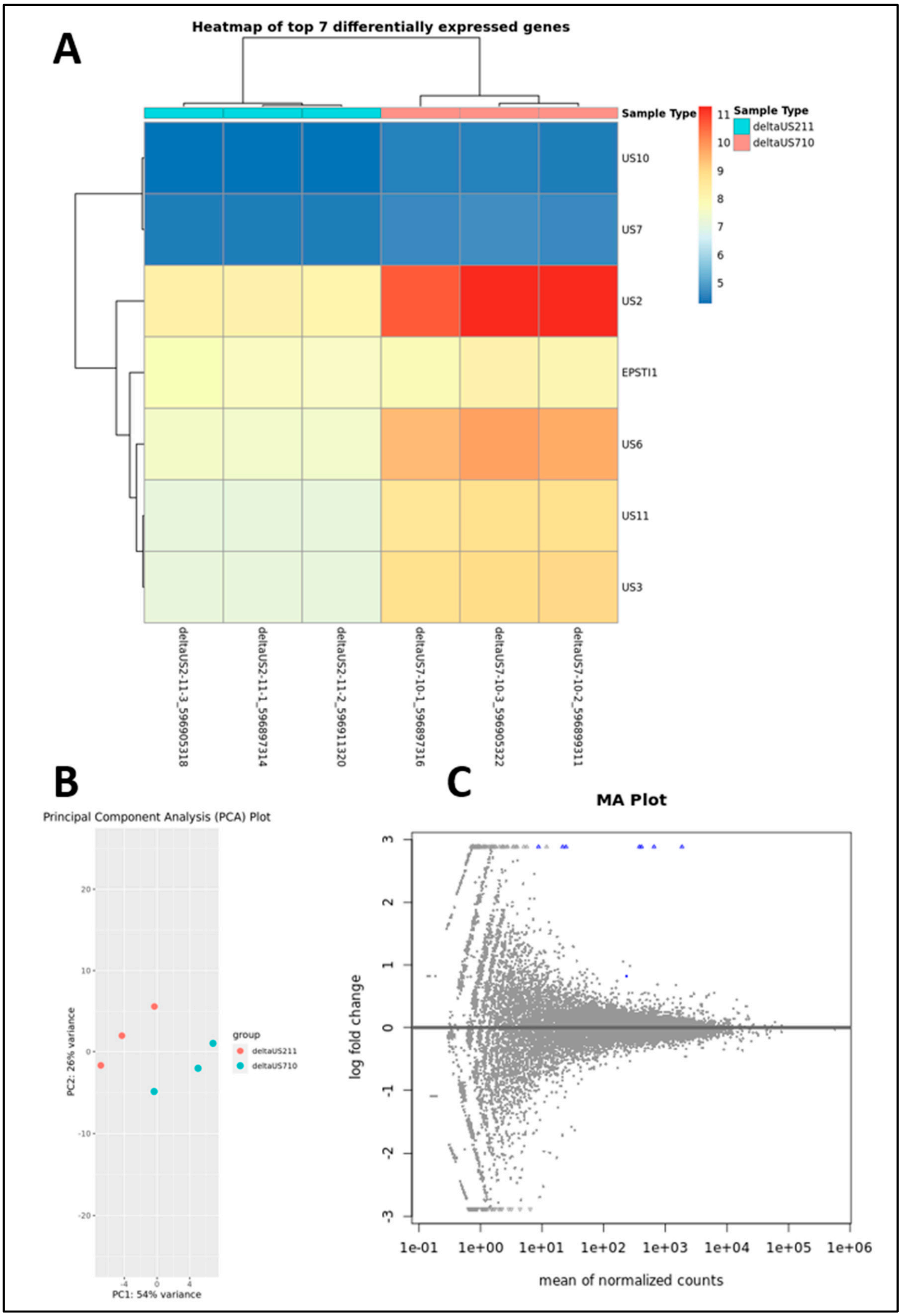

Supplementary Figure S1 – BAD-ΔUS2-11_vs_BAD-ΔUS7-10

RNA-Seq analysis of BAD-ΔUS7-10 in comparison to BAD-ΔUS2-11. (a) Heatmap with expression values for the six samples for all genes with significant signs of differential expression. (b) Principal component analysis for the six samples. (c) Scatter plot of differential gene expression analysis. Each point represents one gene with the x-axis showing the mean of normalized counts and the y-axis indicating the log fold change. Genes significantly up- and downregulated are highlighted in blue (the arrows at the figure's margins point to outliers).
